# Optimal dispersal in ecological dynamics with Allee effect in metapopulations

**DOI:** 10.1371/journal.pone.0218087

**Published:** 2019-06-20

**Authors:** Marcelo A. Pires, Sílvio M. Duarte Queirós

**Affiliations:** 1 Centro Brasileiro de Pesquisas Físicas, Rio de Janeiro/RJ, Brazil; 2 National Institute of Science and Technology for Complex Systems, Rio de Janeiro/RJ, Brazil; Universitat Rovira i Virgili, SPAIN

## Abstract

We introduce a minimal agent-based model to understand the effects of the interplay between dispersal and geometric constraints in metapopulation dynamics under the Allee Effect. The model, which does not impose nonlinear birth and death rates, is studied both analytically and numerically. Our results indicate the existence of a survival-extinction boundary with monotonic behavior for weak spatial constraints and a nonmonotonic behavior for strong spatial constraints so that there is an optimal dispersal that maximizes the survival probability. Such optimal dispersal has empirical support from recent experiments with engineered bacteria.

## Introduction

The Allee effect is an influential finding named after the ecologist Warder Clyde Allee [1] concerning a phenomenon typically manifested by the departure from the standard logistic growth that enhances the susceptibility to extinction of an already vulnerable sparse population. Curiously, W. C. Allee did not provide a definition of the effect [2], but in general terms it can be defined as “the positive correlation between the absolute average individual fitness in a population and its size over some finite interval”. [3]. The strong Allee effect, which is the focus of this work, corresponds to the case when the deviation from the logistic growth includes an initial population threshold below which the population goes extinct [4]. On the other hand, there exists a weak version of the Allee effect which treats positive relations between the overall individual fitness in the population density and does not present population size nor density thresholds.

The Allee effect can emerge from a variety of mechanisms such as mate limitation, cooperative breeding, cooperative feeding, habitat amelioration [3, 4]. Empirical support to the Allee Effect can be find in terrestrial arthropods, aquatic invertebrates, mammals, birds, fish, and reptiles [4, 5]. In addition, thanks to Synthetic Biology it is possible to observe the Allee effect in programmed bacteria [6].

Besides ecology, conservation biology [4] and invasion biology [7], there is a growing number of studies addressing the importance of the Allee effect in other subjects such as epidemiology [8–10] and cancer biology [11, 12] among others. Explicitly, in Ref. [11] the authors suggest the manifestation of the Allee effect as the tumor growth threshold may be explored in therapeutics.

For long the Allee effect was mostly studied at the population scale, but in Ref. [13] it was shown its relevance at the metapopulation level as well. Afterwards, it was effectively demonstrated the Allee effect at the metapopulation level can come up from the Allee effect at the local population level [14, 15].

Focussing on the theoretical approach to the problem, several models—spanning from phenomenological to purely microscopic proposals—have been able to reproduce the Allee effect and to explore its dynamical outcomes [7, 16, 17], namely those coping with the interplay between the Allee effect and dispersal. Note that Depending on the primary approach to population dynamics, the concept of dispersal is also known as migration or diffusion. Let us mention a few examples: on the one hand, one can find works showing a positive association between migration and the number of invaded patches [18]; the invasion diagram presented in Ref. [19] shows that the propagation failure regime shrinks as the dispersal rate increases. In Ref. [20], it is asserted that in a simple metapopulation dynamics the larger the migration the larger the mean time to extinction. On the other hand, there are works indicating that the combination of the Allee effect and dispersal produces a negative impact on the population dynamics; that is the case of Ref. [21] where the authors claim that the vulnerability to extinction increases with the mean-square displacement. Considering a nonlinear dynamics analysis of the Allee effect, the survival-extinction bifurcation diagram shown in Ref. [22] reveals that the extinction regime augments directly with the dispersal probability. Complementary, it was also found that a dispersive population under the Allee effect faces a dramatically slowed spreading [23]. Additionally, it was shown in [24] that the dispersal does not always enhance regional persistence in a predator-prey system under the Allee effect. Last, the results conveyed in Ref. [25] indicate that populations with the Allee effect face an inverse relationship between the settlement probability and the pre-mating dispersal.

Particularly in population ecology, Windus and Jensen [26] proposed a minimal model that successfully captures the Allee Effect by means of a bistable dynamics arising from microscopic rules. Inspired by their model, we develop an ecological metapopulation dynamics in order to explore how the threefold interplay between the Allee Effect, dispersal and spatial constraints impacts on the survival probability of a population dynamics. It is reasonably expected that the dispersal has a beneficial impact on population survival by decreasing the local competition for resources. But interestingly, we observe that for severe spatial constraints there is the emergence of an optimal dispersal rate that promotes the highest survival probability. This nonmonotonic relation between survival and dispersal—which is not very intuitive at first glance—was recently observed in controlled experiments with engineered bacteria [6].

## Materials and methods

Consider a metapopulation [27, 28] with *L* subpopulations composed of agents that are able to move, die or reproduce. As usual in metapopulation dynamics [27], we assume a well-mixed subpopulation, i.e., inside each subpopulation all individuals have the possibility to interact one another. In Statistical Physics parlance that is to say that our local dynamics exhibits a mean-field character. The mobility is implemented as a random walk between neighbor subpopulations such that migration occurs at each time step with probability *D*. At a given time step, if migration does not take place (with probability 1 − *D*) then one of the two events is chosen [26]: death of an agent with probability *α* or reproduction with probability λ when two agents meet. Mating limitation is an important source of the Allee effect [25, 29] which in our model is incorporated in the reproduction event.

At this point, three remarks are worth making: first, heed that *D* controls the difference of time scale between the patch mobility and the reproduction/death; second, we make no extra assumptions on the probabilities *α* or λ; and third, this proposal naturally incorporates the environmental changeability seeing that the carrying capacity of each subpopulation is not fixed. Moreover, there is no local condensation of the agents because the random walk uniforms the agents distribution among the subpopulations.

We would like to stress that our goal is not to model specific ecological dynamics, but to investigate the possible emerging scenarios from this minimal agent-based migration-reproduction-death dynamics instead. This approach can be seasoned with further elements that account for the traits of a given system. It is well-known that the use of minimal models is very helpful in providing an understanding of the cornerstone mechanisms present in tailored models.

### Monte Carlo algorithm

Computationally, we use an array with *N* states divided into the *L* subpopulations. Each state in subpopulation *u* indicates an agent, $i_{A}^{u}$ or a vacancy, $i_{V}^{u}$. Our time unit is a Monte Carlo step (mcs) that consists of a visit to each one of the *N* states. Our main code is available at [30].

**Monte Carlo Step**:

For each state *i* = 1, …, *N*:

- First get the subpopulation, say *u*, of the state *i*.
- With probability *D*:
  - **Dispersal**: If the state *i* indicates an agent, $i_{A}^{u}$, then move it to one of its neighbors *w* chosen at random: $i_{A}^{u}\Rightarrow i_{A}^{w}$. (event 1)
- With probability 1 − *D*:
  - **Reproduction**: If the state *i* indicates a vacancy, $i_{V}^{u}$, then pick at random another state *j* in the same subpopulation *u*. If this *j* indicates an agent, $j_{A}^{u}$, then pick at random another state *l* in the same subpopulation *u*. If the state *l* indicates another agent, $l_{A}^{u}$, then transform the vacancy $i_{V}^{u}$ into an agent $i_{A}^{u}$ with rate λ: $i_{V}^{u}+j_{A}^{u}+l_{A}^{u}\Rightarrow i_{A}^{u}+j_{A}^{u}+l_{A}^{u}$. (event 2)
  - **Death**: If the state *i* indicates an agent, $i_{A}^{u}$, then transform it into a vacancy with rate *α*: $i_{A}^{u}\Rightarrow i_{V}^{u}$. (event 3)

After each mcs apply a synchronous update of the states.

### Analytical considerations

Consider that *A*_*u*_(*t*) and *V*_*u*_(*t*) are the number of agents and vacancies in the subpopulation *u* at time *t*, with corresponding local fractions *a*_*u*_ = *A*_*u*_/(*V*_*u*_ + *A*_*u*_) and *v*_*u*_ = *V*_*u*_/(*V*_*u*_ + *A*_*u*_), respectively. Let *u* = 1, …, *L*. We consider a circular/ring metapopulation where each node is a subpopulation connected to *k* neighbor subpopulations. That parameter controls the magnitude of the spatial constraints, i.e., *k* means the number of new pathces that individuals can move to from its current patch.

Taking into account the 3 events described in our algorithm and considering the well-mixed population (mean-field) at the local scale, the rate of change of *v*_*u*_ and *a*_*u*_ are given by

$$dv_{u}/dt=\left(1-D\right)\left(p_{death}-p_{rep}\right)$$ (1)

and

$$da_{u}/dt=\left(1-D\right)\left(p_{rep}-p_{death}\right)+D\left(-p_{out}+p_{in}\right)$$ (2)

respectively. The effective probability of successful reproduction of two individuals in an available vacancy is $p_{rep}=\lambda v_{u}a_{u}^{2}$. The effective probability of death of one individual depends on the fraction of individuals at a given subpopulation and the rate of death, *p*_*death*_ = *αa*_*u*_. The probability of selecting an individual to move out of a given subpopulation *u* is just *p*_*out*_ = *a*_*u*_. Finally, the probability of individuals in the neighbors *z* moving into *u* is proportional to the fraction of individuals in neighbors *a*_*z*_ weighed by the number of possible choices of movement *k*, $p_{in}=\sum_{z=1}^{L}\frac{1}{k}W_{uz}a_{z}$, with *W*_*uz*_ being the elements of the adjacency matrix which assumes the value 1 if *u* and *z* are connected or 0 otherwise. Considering all these terms we can rewrite the dynamical equations in terms of *A*_*u*_(*t*) and *V*_*u*_(*t*) to obtain the equations ruling the overall system

$$\frac{dV_{u}}{dt}=\left(1-D\right)[\overset{\text{Reproduction}}{\overset{︷}{-\frac{\lambda V_{u}A_{u}^{2}}{{\left(V_{u}+A_{u}\right)}^{2}}}}+\overset{\text{Death}}{\overset{︷}{\alpha A_{u}}}]$$ (3)

$$\frac{dA_{u}}{dt}=\left(1-D\right)[\underset{\text{Reproduction}}{\underset{︸}{\frac{\lambda V_{u}A_{u}^{2}}{{\left(V_{u}+A_{u}\right)}^{2}}}}-\underset{\text{Death}}{\underset{︸}{\alpha A_{u}}}]+D[\underset{\text{Emigration}}{\underset{︸}{-A_{u}}}+\underset{\text{Immigration}}{\underset{︸}{\sum_{z=1}^{L}\frac{1}{k}W_{uz}A_{z}}}]$$ (4)

Aiming at taking into account both the cases of single and multiple sources of invasion, we shall use an initial condition given by

$$A_{u}\left(0\right)=\{\begin{array}{cc} \frac{1}{n_{s}}\frac{N}{L} & u=1,\ldots ,n_{s}\\ 0 & u=n_{s},\ldots ,L \end{array}$$ (5)

where *N*/*L* is the initial size of each subpopulation and *n*_*s*_ is the number of initial sources. By default, we use *V*_*u*_(0) = *N*/*L* − *A*_*u*_(0) as well.

### Survival-extinction phase transition

From a preliminary numerical analysis we observed that the steady-state solution satisfies

$$A_{u}^{\infty }=\bar{A},\;V_{u}^{\infty }=\bar{V}\;\forall u\;u=1,2,\ldots ,L$$ (6)

which can be used as an ansatz to our problem yielding

$$N=\sum_{u=1}^{L}\left(A_{u}+V_{u}\right)=L\left(\bar{A}+\bar{V}\right)\Rightarrow \bar{V}=N/L-\bar{A}$$ (7)

$$\frac{d\bar{A}}{dt}=\left(1-D\right)[\frac{\lambda \left(N/L-\bar{A}\right){\bar{A}}^{2}}{{\left(N/L\right)}^{2}}-\alpha \bar{A}]+D[-\bar{A}+\frac{1}{k}(k\bar{A})]=0$$ (8)

Then from Eq (8) we obtain three solutions to $\bar{A}$. The stability analysis provides an overall picture of the steady-state

$$A_{u}^{\infty }=\{\begin{array}{cc} \frac{N}{2L}(1\;+\sqrt{1-4\frac{\alpha }{\lambda }}) & A_{u}\left(t=0\right)\geq A_{c}^{o}\;\text{and}\;\alpha \geq \lambda /4\\ 0 & \text{otherwise} \end{array}$$ (9)

where $A_{c}^{o}$ is the threshold initial population size required for the local persistence:

$$A_{c}^{o}=\frac{N}{2L}(1-\sqrt{1-4\frac{\alpha }{\lambda }})$$ (10)

Eqs (9) and (10) do not explicitly take into account the dispersal parameter *D*, but they allow us to get an insight into the nature of the survival-extinction phase transition: they show that the subpopulation faces a discontinuous transition at the critical point *α*_*c*_ = λ/4. This discontinuous transition is verified for the dynamics of the whole system inasmuch as the mobility of the agents induces the long-run absence of local correlations to the whole metapopulation.

## Results and discussion

In this section, we present the results for metapopulations of sizes 10 ≤ *L* ≤ 50 and increasing *k*. By means of Monte Carlo simulation and bearing in mind Eqs (1) and (2) we confirmed that the results are still valid for larger networks that correspond to the limit of a macroscopic system (the so-called thermodynamic limit). For the sake of simplicity and without loss of generality the results we show are for λ = 1.

**Fig 1:**
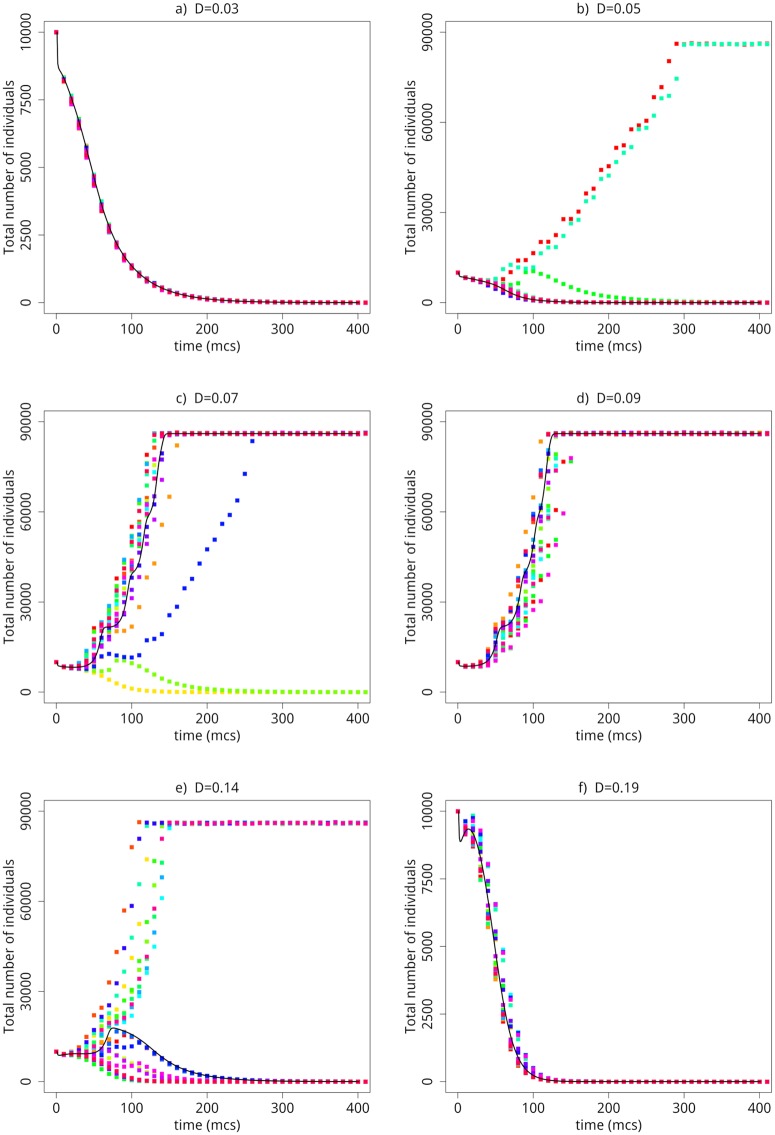
Time series (in mcs) for the total number of of agents for *D* = {0.03, 0.05, 0.07, 0.09, 0.14, 0.19} with *L* = 10, *N* = 10^4^ *L*, *n*_*s*_ = 1 and *k* = 2. Each color corresponds to one sample. The symbols were obtained from Monte Carlo simulations and the lines from Eqs (3) and (4).

**Fig 2:**
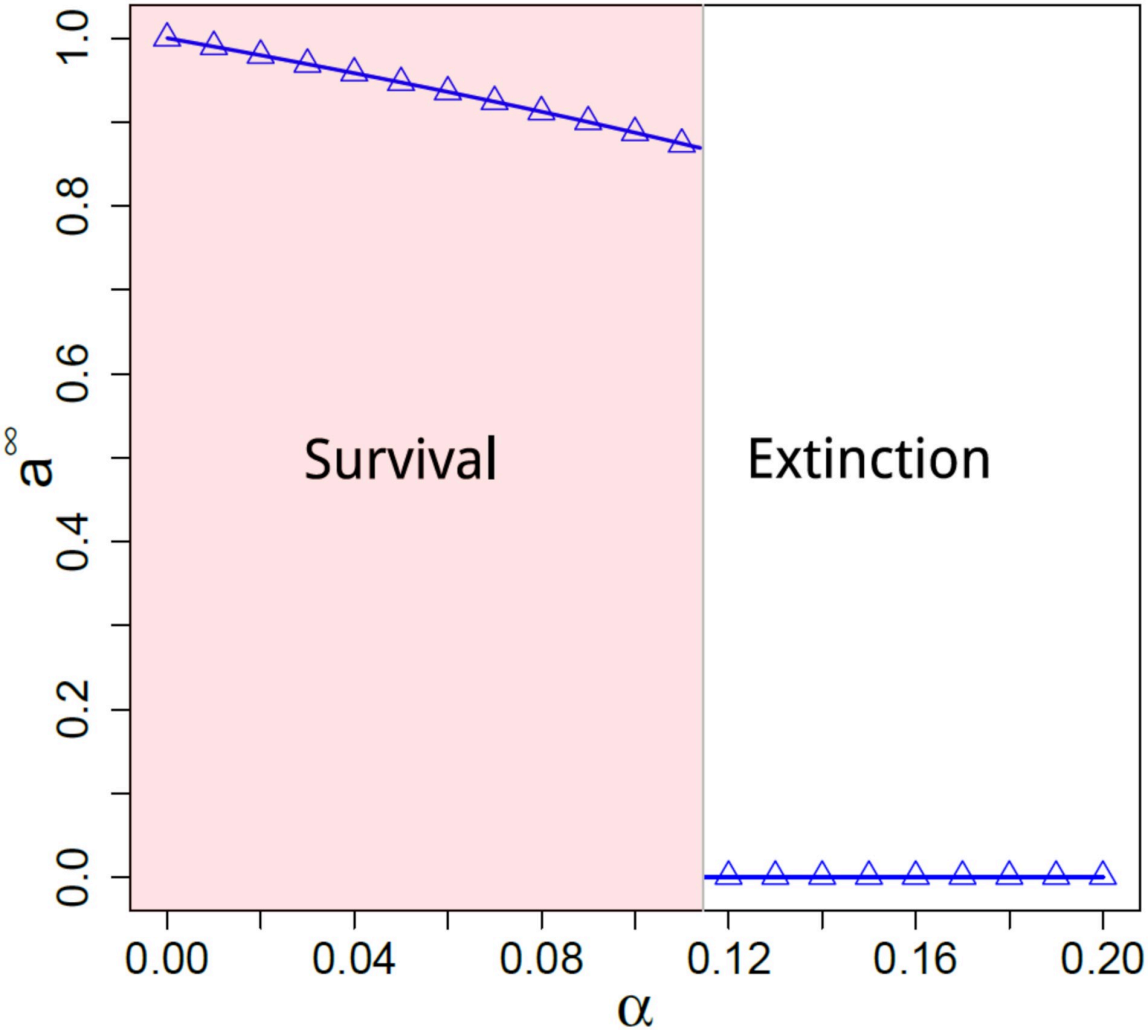
Stationary density of agents *a*^∞^ vs mortality rate *α* with *D* = 0.2, *k* = 2, *L* = 10, *N* = 10^4^ *L*, *n*_*s*_ = 1. The symbols come from the Monte Carlo Simulations and the lines come from the numerical integration of Eqs (3) and (4).

Looking at the time series of the total number of agents in the metapopulation for different dispersal rates as depicted in Fig 1. The evolutions for *D* = {0.03, 0.09, 0.019} exhibit a single stable (steady) state; however, the cases with *D* = {0.05, 0.07, 0.014} display bistable solutions. This rich dynamics is the outcome of the competition between reproduction (event 2) and mortality (event 3). It is worth stressing the role of randomness—governed by our probability parameters—on the development of that bistability. The combination of randomness and bistability paves the way to ecological scenarios in which extinction can take place with no apparent reason, even in the presence of abundant resources whatsoever. Last, the scenario marked by two well-separated stochastically-induced steady-states is the hallmark of a sudden phase transition that we depict in Fig 2; therein, one perceives the density of individuals—which is our order parameter—displays a sheer increase of a critical mortality rate *α*_*c*_. To fully grasp the idea behind the survival-extinction transition in Fig 2, we consider the different ecological scenarios corresponding to *α* = {0.04, 0.08, 0.12}. Explicitly, if we increase *α* = 0.04 to *α* = 0.08—ie, environmental conditions for mortality are enlarged—the total density of individuals undergoes just a slight drop. However, if the mortality parameter *α* becomes larger than *α* = 0.08 (eg, *α* = 0.12), there is a significant dynamical response in the population density, specifically the mass extinction. That is to say, an augment in the mortality rate can either spark a small or a drastic decline in the population. That means that depending on the proximity to the threshold point the population can exhibit a robust profile with respect to environmental perturbations or else behave in a quite vulnerable way. This feature is a remarkable fingerprint of a discontinuous phase transition. It is worth mentioning that abrupt phase transitions are not an odd phenomenon in biological dynamics [31].

**Fig 3:**
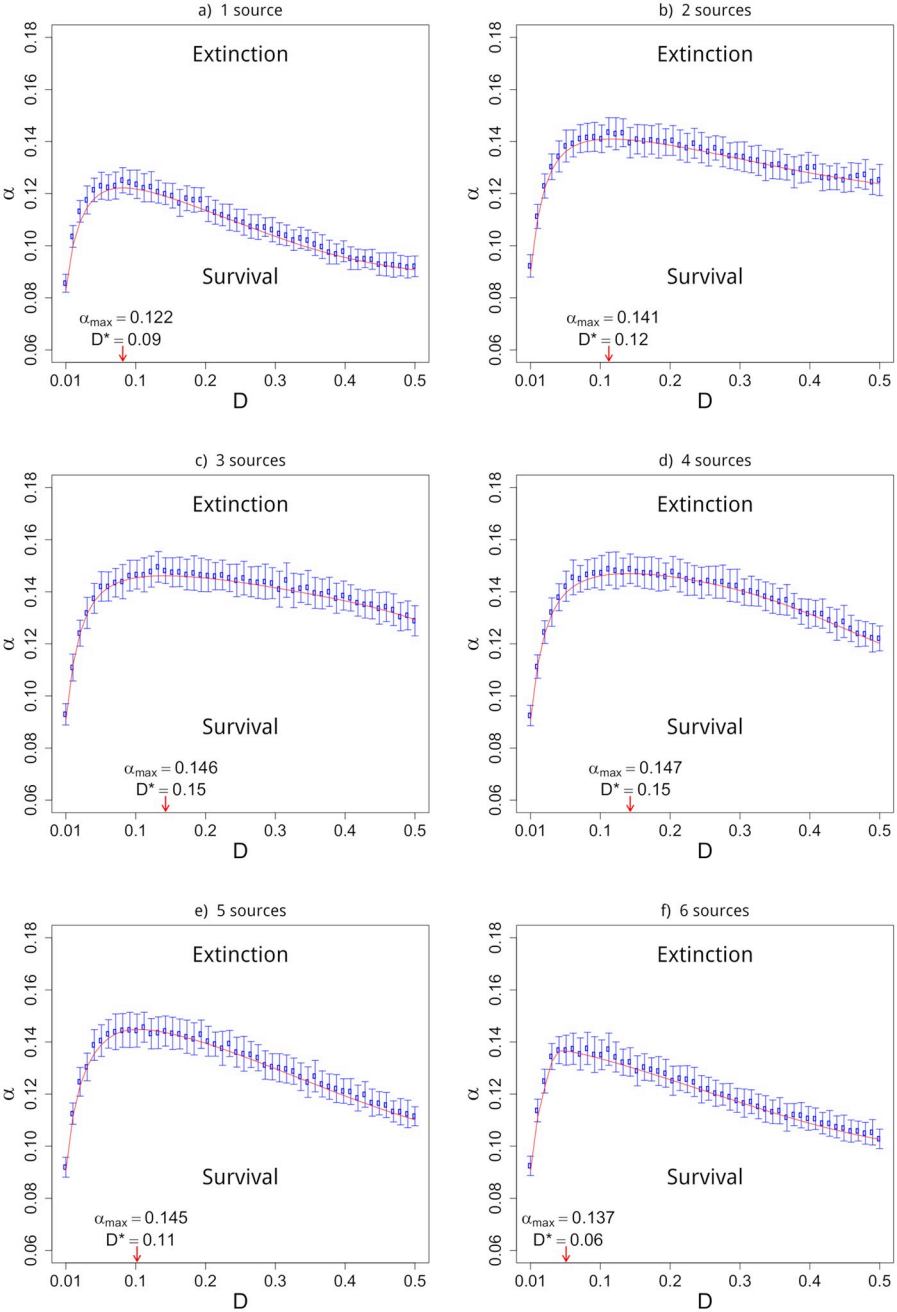
Phase diagram *α* v *D* for *n*_*s*_ = 1, 2, …, 6 sources, *L* = 10, *N* = 10^4^ *L* and *k* = 2. The point *D* = 0 is excluded from the diagram since it refers to isolated populations with threshold *α*_*c*_ = λ/4 = 0.25. In all the cases $n_{0}=\frac{10^{4}}{n_{s}}$, where *n*_0_ is the initial subpopulation size. The lines are obtained from Eqs (3) and (4). At the bottom of each plot we show the optimal dispersal D* and the corresponding maximum allowed mortality rate *α*_*max*_(D*) below which the population still stay in the survival phase.

Up to now, we have not separated out the roles played by *D* and *n*_*s*_ on the threshold *α*_*c*_(*D*). To that, we call attention to Fig 3 where it is possible to disentangle the effect produced by each parameter. Resorting to an iterative procedure close to that described in section 2.1 of Ref. [26]: (i) set an initial guess for the threshold *α*_*c*_′, and start the algorithm; (ii) if a sample reaches extinction, the seed *α*_*c*_′ is decreased by *dα*; (iii) if a sample shows a long-term persistent population, then *α*_*c*_′ is increased by *dα*. The results of this process are plotted in Fig 3 where is possible to verify a good agreement with the theoretical threshold obtained from Eqs (3) and (4). Heed there is an optimal dispersal rate D* that leads to the maximum allowed mortality rate *α*_*max*_ below which the population will be within in the survival phase. Thus, a population with an optimal dispersal rate D* is less likely to enter the extinction phase than populations with sub-optimal dispersal *D* = D* ± *δ* for *δ* > 0. In other words, in Fig 3 it is clear that the optimal dispersal allows the population to stay in the survival phase with mortality rates that leads other populations with *D* = D* ± *δ* (faster/slower) to the fate of extinction. In respect of the number of sources, we verified that it does not change the qualitative nonmonotonic dependence of *α* vs *D*, only the magnitude of the dependence is sensitive to that.

**Fig 4:**
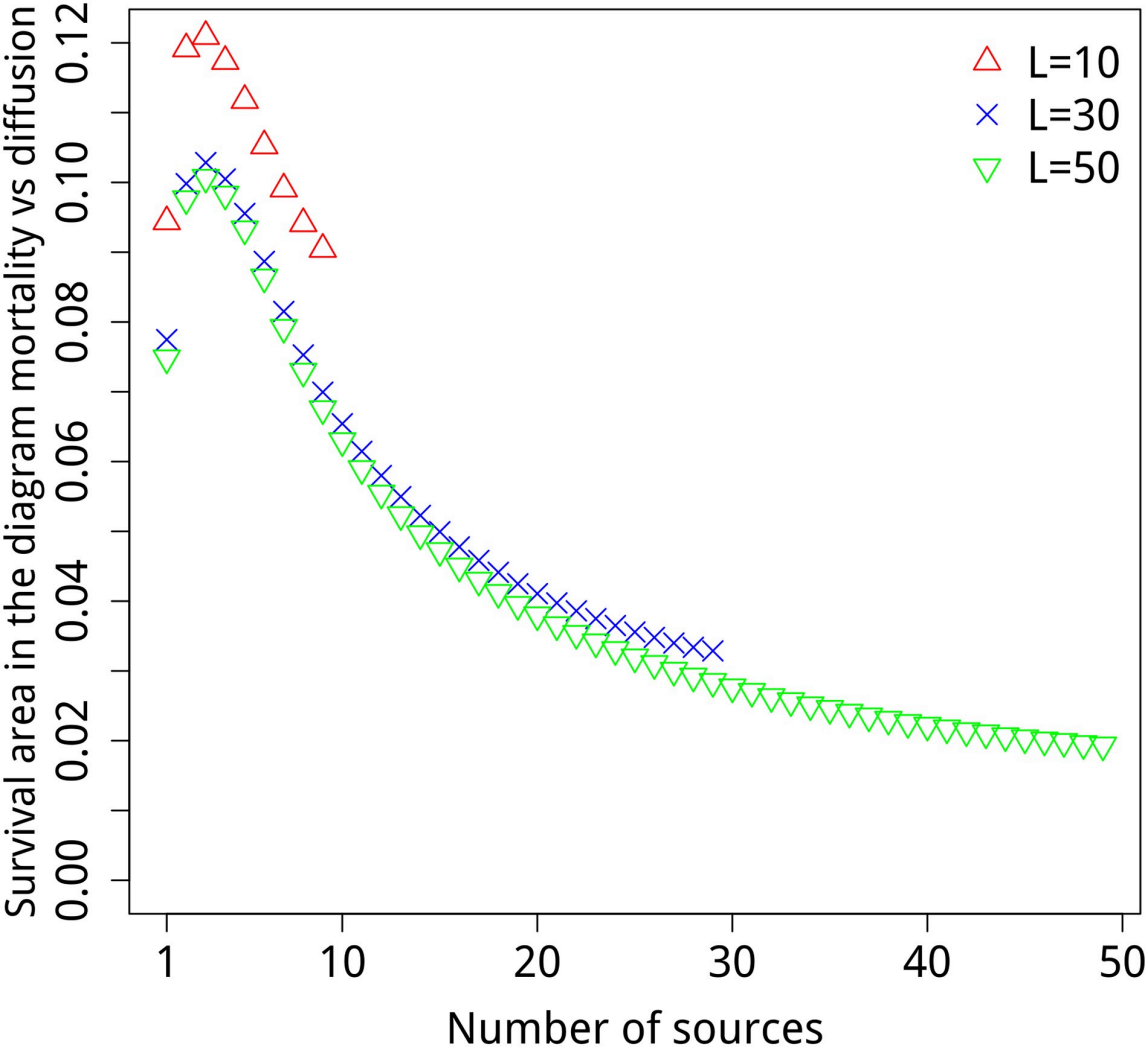
Survival area in the phase diagram *α* × *D* versus the number of sources *n*_*s*_ for 0 < *D* < 1. The case *D* = 1 is excluded because it implies no reproduction/death. The case *D* = 0 is excluded because it implies no migration between the patches. In all cases we keep the initial subpopulation size fixed $n_{0}=\frac{10^{4}}{n_{s}}$ and we use *N* = 10^4^ *L*.

Interestingly, Fig 4 shows there is an optimal number of sources that promotes the largest survival area in the diagram *α* vs *D* > 0, as anticipated in Fig 3. This means that the survival probability is maximized for an intermediate number of sources, wherefrom we understand that in populations subjected to the Allee Effect it is best to spare the population in many sources, but not too much. Similar results were found in Ref. [15] where the authors came up with an integrated model that displays an Allee-like effect at the metapopulation level, which is the outcome of imposing the Allee effect at the local population level. That is in contrast with our work because we use a microscopic model with no extra assumption on birth and death rates.

**Fig 5:**
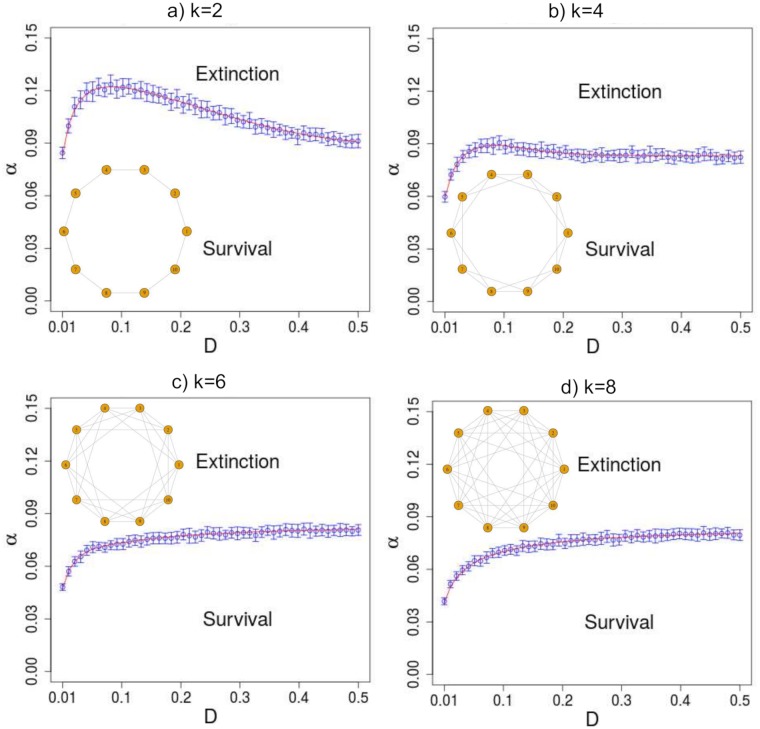
Phase diagram *α* vs *D* for networks with increasing number of neighbors *k* = 2, 4, 6, 8 (decreasing spatial constraints). The theoretical lines (red) comes from numerical integration of Eqs (3) and (4). The optimal dispersal and the corresponding maximum *α* are: (a) D* = 0.09 and *α*_*max*_ = 0.122; (b) D* = 0.092 and *α*_*max*_ = 0.089.

**Fig 6:**
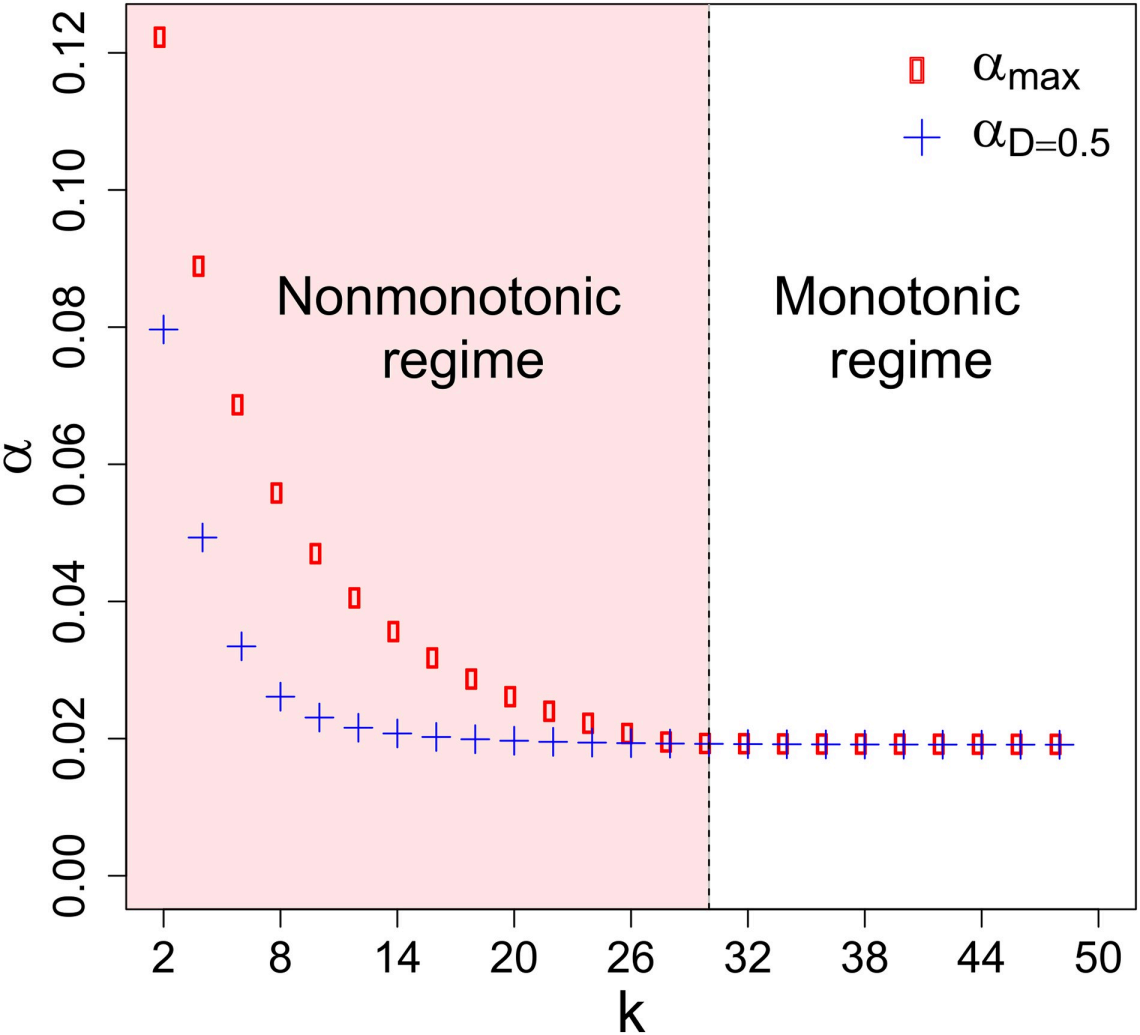
Regime diagram of the dependence between threshold mortality *α*_*c*_ vs dispersal rate *D* for *L* = 50. The vertical line that separates the two regimes is *k*_*threshold*_ = 30. For *k* < *k*_*threshold*_: *α*_*max*_ > *α*_*D* = 0.5_ then *α*_*c*_ × *D* displays a nonmonotonic dependence. For *k* ≥ *k*_*threshold*_: *α*_*max*_ = *α*_*D* = 0.5_ then *α*_*c*_ × *D* exhibits a monotonic dependence.

The survival-extinction phase diagram in Fig 5 shows that a decrease in the severity of the spatial constraints—i.e., an increase of *k*—leads to a decreasing in the threshold mortality *α*_*c*_(*D*) for all *k*. That is to say, the population becomes more vulnerable to extinction when there are more open paths to emigrate. This result goes along the finding in Ref. [18] where it was found that a decreasing in the number of connections enhances the invasion probability. Furthermore, we observe the emergence of two different regimes: *α*_*c*_ increases nonmonotonically with *D* for severe spatial constraints (*k* = 2, 4), but it increases monotonically with *D* for loose spatial constraints (*k* = 6, 8). Although we used a simplified minimal network it already shows the importance of spatial constraints in changing the qualitative behavior of the system. At last, Fig 6 summarises our results for different magnitudes of spatial constraints *k*. Clearly, there is a threshold for *k*, above which there is a monotononic dependence between *α*_*c*_ and *D*.

So, what is the underlying mechanism behind the qualitative change presented in Figs 5 and 6? First, let us keep in mind that the Allee effect in our approach is manifested by the mate-finding limitation. When the geometric constraints are severe, we have a nonmonotonic regime caused by the source-sink dynamics between the donor subpopulation and its surroundings. For small dispersal, the source cannot provide enough individuals to produce a sustainable colony in the first-neighbors that in turn acts as a drain from the donor subpopulation. For intermediate dispersal, the first neighbors receive enough individuals to bear sufficient reproduction to overcome the Allee Effect. Nonetheless, if the dispersal is further augmented, then the first neighbors receive as many individuals as they lose to their next-nearest neighbors, which yields an insufficient net reproduction to guarantee long-term survival. Alternatively, in the monotonic regime, the loose spatial constraints allow the emergence of multiple secondary sources that feed one another in a way that, in boosting the dispersal, one enhances the net reproduction to surpass the Allee effect.

From an empirical perspective, the work by Smith et al [6] supports our finding regarding optimal dispersal. Therein, they engineered E. coli colonies aiming at displaying the strong Allee effect and found that the dispersal acts as a double-edged sword. In other words, intermediate dispersal rates favor bacterial spreading whereas both low and high dispersal rates inhibit it. Additionally, they provide empirical evidence for another result of ours in Figs 5 and 6: increasing connectivity can increase the vulnerability to extinction.

In spite of the fact that it is known that reaction-diffusion equations with a linear population growth can exhibit a critical diffusion rate (for a given habitat size) guaranteeing the survival of the population [33], we would like to emphasize that our work goes beyond that: we have shown that the relationship between the survivability and dispersal undergoes a transition of monotonicity when a birth-death process with the Allee effect is subjected to the interplay between dispersal and tunable spatial constraints.

## Conclusions

In this work, we have investigated the spectrum of scenarios arising from a metapopulation dynamics under the Allee Effect using a minimal agent-based model which points at describing fundamental mechanisms thereof. Employing numerical and analytical tools we have shown that the survival-extinction boundary undergoes a monotonicity transition: it has a nonmonotonic behavior marked by an optimal dispersal for severe spatial constraints, but a monotonic behavior for loose spatial constraints. The verification of this qualitative change in the dependence of the mortality threshold as a function of the dispersal highlights the importance of the triangular interplay between the Allee Effect, dispersal and geometric constraints for the persistence of populations.

Besides the experimental work of Ref. [6], there are other previous theoretical models pointing to the same conclusions over the likely existence of an intermediate mobility rate that optimizes the survival probability. Explicitly, in Ref. [32] the authors found a nonmonotonic relationship between the critical Allee threshold and the migration parameter by imposing the Allee Effect at the microscopic scale which is made considering a nonlinear per capita birth rate *rn*_*i*_/*C* + *rn*_*i*_ *c*/*C*^2^ and per capita death rate $rn_{i}^{2}/C+rc/C$. where *n*_*i*_ stands for the number of individuals on habitat patch *i*, *C* is the carrying capacity, *c* is an Allee threshold. In addition, we can refer to the results in Ref. [34] in which it was used an individual two-gender population on a hexagonal grid where the juveniles disperse away from their natal territory with dispersal distances distributed as a negative exponential. In that case, the population growth was highest for an optimal distance of the dispersal. In [35] they found that the dispersal can have antagonistic effects on the persistence of biological control introduced under the Allee effect. However, none of these studies have found the major novelty of our work: increasing the magnitude of the spatial constraints can change qualitatively the survival-extinction boundary from a nonmonotonic to a monotonic dependence. Our finding prompts an inquiry into the actual role of the network topology [36, 37] in the macroscopic outcome of ecologic dynamics; something we intend to explore in future work.

In a broader view, there are other biological systems that exhibit nonmonotonic effects of dispersal such as epidemic spreading [38], birth-death-competition dynamics with migration [39], evolutionary dynamics with the Allee effect and sex-biased dispersal [40], logistic growth dynamics in metapopulations with heterogeneous carrying capacities [41], metapopulation genetics dynamics with balancing selection [42], two-type (mutants, strains, or species) population dynamics under the Allee effect [43], and range expansion of a genetically diverse population where individuals may invest its limited resources partly in motility and partly in reproduction [44]. As we adopted a minimal ecological model, it is possible to bring forth different extensions of the present work in order to fit for the traits of the problems aforementioned For instance, instead of using a memoryless random walk, we can use a more realistic mobility dynamics: random walks that intermittently revisits previously visited places [45].
